# *Hibiscus sabdariffa*: Genetic variability, seasonality and their impact on nutritional and antioxidant properties

**DOI:** 10.1371/journal.pone.0261924

**Published:** 2022-03-16

**Authors:** Abdoudramane Sanou, Kiessoun Konate, Roger Dakuyo, Kaboré Kabore, Hemayoro Sama, Mamoudou Hama Dicko

**Affiliations:** 1 Department of Biochemistry and Microbiology, Laboratory of Biochemistry, Food Biotechnology and Nutrition, University Joseph KI-ZERBO, Ouagadougou, Burkina Faso; 2 Applied Sciences and Technologies Training and Research Unit, University of Dedougou, Dedougou, Burkina Faso; 3 Laboratory of Biochemistry and Chemistry Applied (LABIOCA), University Joseph KI-ZERBO, Ouagadougou, Burkina Faso; Gachon University, REPUBLIC OF KOREA

## Abstract

This study consisted of the physicochemical, phytochemical and antioxidant characterisation of two varieties of *Hibiscus sabdariffa*, to evaluate the influence of genetic and environmental factors on these parameters leading to an objective and rigorous classification of our extracts. To this end, calyxes and seeds of the red and white phenotypes were collected in Bobo-Dioulasso, Dano and Nouna respectively in December 2019 and 2020. Principal component analysis showed that physico-chemical and biochemical parameters could potentially be used to discriminate varieties. The calyxes of the *sabdariffa* variety showed the best physicochemical profile (total phenolics, flavonoids, ascorbic acid), pigments (anthocyanins, chlorophyll) and antioxidant activity (free radical scavenging and SOD activity) while the seeds showed the best carbohydrate, lipid and peroxidation inhibition content. In view of these results, the red phenotype has an interesting nutritional and therapeutic potential. It could therefore be interesting candidate in the pharmaceutical and food industries.

## 1. Introduction

*Hibiscus sabdariffa L*.) (roselle) belongs to Malvaceae family. It is known by different synonyms and vernacular names, such as roselle, Indian sorrel, Jamaica sorrel, Guinean sorrel, red sorrel, Mesta and karkade [1]. *H*. *sabdariffa* is also recognized as an important source of value-added compounds such as natural pigments and bioactive compounds whose isolation is of great interest in food and pharmaceutical industries [2]. Indeed, several previous studies have shown that *H*. *sabdariffa* is an important source of. phenolic compounds with nutritional, diuretic, antidiabetic, antilipidemic and antihypertensive properties [3]. In particular, *H*. *sabdariffa* is a powerful hypotensive due to its rich phenolic composition. These polyphenols prevent oxidative stress, reduce thrombosis, oxidative stress, heart disease, endothelial dysfunction, inflammation and modify the expression of genes responsible for the atherosclerosis process [4]. Phenolic compounds are receiving particular attention in fruits and vegetables because of their association with enhanced antioxidant activity in *vitro* and in *vivo*, linked to their ability to scavenge free radicals [5]. Among these phenolic compounds, *H*. *sabariffa* mainly contains organic acids (Hydroxy-citric acid, hibiscus acid), anthocyanins (delphinidin-3-sambubioside, cyanidin-3-sambubioside), flavonoids and phenolic acids (gallic acid, quercetin, kaempherol, caffeic acid, chlorogenic acids, Galloyl ester…) [6]. However, the biosynthesis of these bioactive compounds is largely influenced by genetic and environmental factors (rainfall, temperature, state of maturity and cultivation practices) [7]. These secondary metabolites are synthesized via shikimate pathway. They are involved to plant response to environmental stresses (water stress, salt stress, etc.) which can lead to an uneven concentration and distribution of bioactive substances in the different compartments of the plant. Similarly, secondary metabolites may derive from modified synthetic pathways from a primary metabolite, or from sharing the original substrates of the primary metabolite [8].

Genetic, environmental, ecological and harvesting conditions also influence the biochemical composition (Da-Costa-Rocha et al., 2014). In Burkina Faso, two varieties of *H*. *sabdariffa* are widely cultivated (var *sabdariffa* with red phenotype and var *altrissima* with white one). The calyxes are used in the production of juice and the seeds in the manufacture of bikalga ((alkaline food condiment). The knowledge of the influence of genetic and seasonal variability on the nutritional and therapeutic composition constitutes a guide for the food and pharmaceutical industries for a better valorization of the *H*. *sabdariffa* sector.

## 2. Materials and methods

### 2.1. Plant material

Sample collection was carried out in December 2019 in three cities (Dano, Bobo Dioulasso and Nouna). The biological material consisted of calyces and seeds of each variety of *H*. *sabdariffa* var. *altrissima* and var. *sabdariffa*) and by site. Thus, 90 samples (including 45 samples for calyces and 45 samples for seeds) were respectively collected in Dano (11° 08′ 38″ N, 3° 03′ 43″ W), Bobo Dioulasso (11° 10’ 37 N, -4° 17’ 52 W) and Nouna (12° 43′ 58″ N, 3° 51′ 44″ W). Identification and authentication were carried out by the services of Dr Mohamed CISSE, botanist at the Laboratory of Biology and Ecology of the University Joseph Ki-ZerboTo this end, a reference specimen bearing the number 18015/6975/2020/SA was deposited in the herbarium of the Life and Earth Sciences Unit of the University Joseph Ki-Zerbo. Samples were labelled, stored in a cooler and brought to the laboratory for analysis. A second collection was carried out in December 2020 in order to evaluate the effect of seasonality.

### 2.2. Experimental design and sample preparation

**Fig 1:**
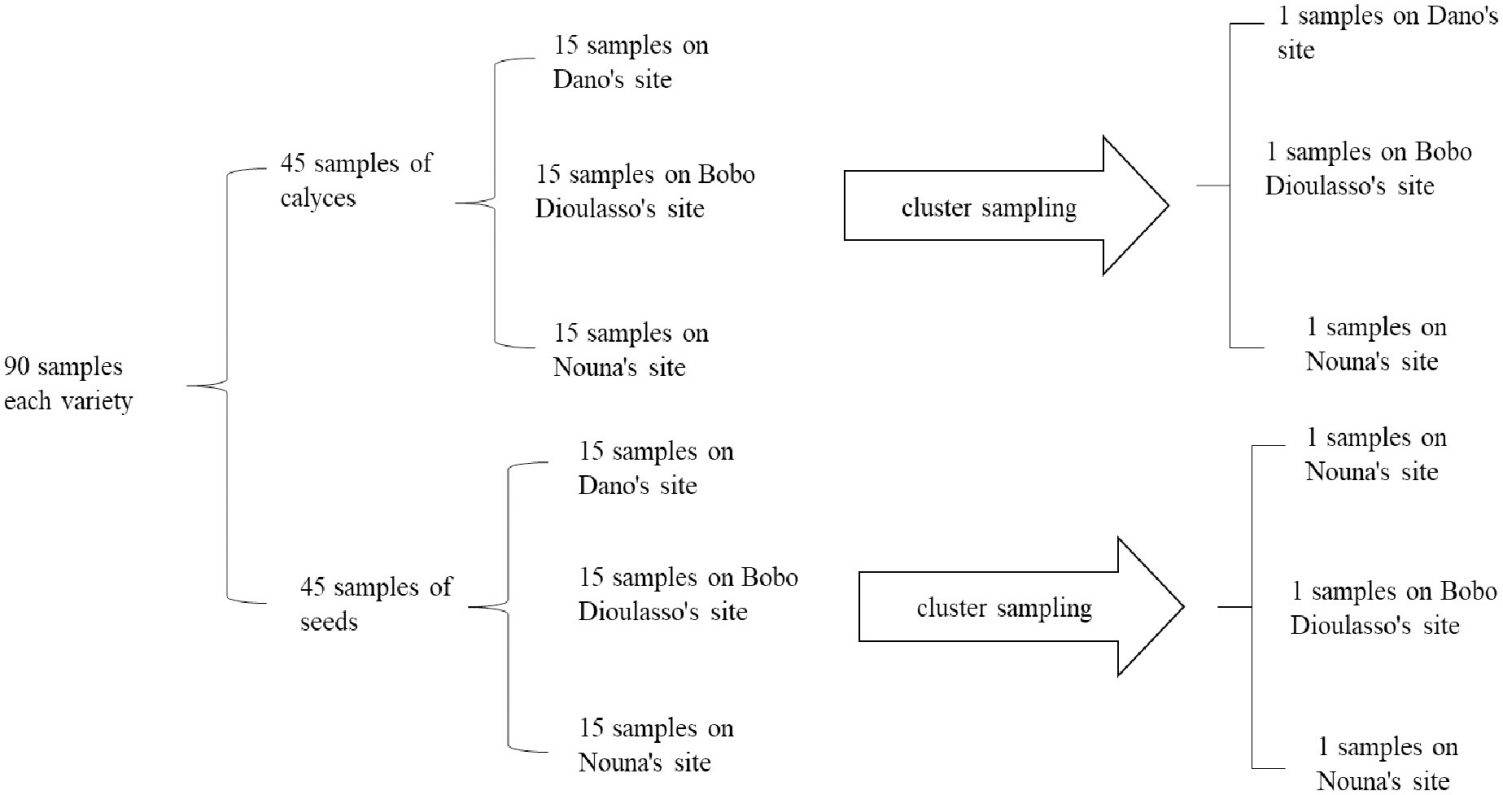
Experimental design.

In order to reduce the influence of cultivation practices and maturity as much as possible, cluster sampling was carried out according to the method cluster sampling described by Taherdoost and al., 2016 [9]. Thus, 12 samples were taken, 6 for calyxes and 6 for seeds, from 180 samples taken from the growing media (Fig 1). After the sampling plan, all samples were sorted, crushed and the extraction of bioactive compounds was performed using ultrasound (Branson 1510) followed by freeze-drying (Biobase). The samples were annotated according to the codes below and stored for one week for the different analyses: CS: calyces of the red phenotype; GS: seeds of the red phenotype, CA: calyxes of the white phenotype and GA: seeds of the white phenotype.

### 2.3. Physical and proximate composition

#### 2.3.1. Determination of water content, pH, titratable acid, dry matter, ash and colour

The analyses for water content and total minerals were carried out according to standard of Association Française de Normalisation (AFNOR) [10]. Water content was determined by drying at 105°C for 24 h (NF V 03–707). Total minerals were determined after 6 hours incineration at 550°C (NF V 76–005). Aqueous extracts of *H*. *sabdariffa* (10 mg/mL) were used for the evaluation of tritrable acidity and pH. The titratable acid of the sample was determined by titration with NaOH 0.01 N and values were expressed in milligram equivalent potassium hydroxide per 100 grams (mgEKOH/100g). pH values were measured using a pH meter (PHS-25CW Microprocessor instruments). The colour analysis of samples was performed with a colorimeter (PCE-CSM1 colorimeter) according to the colour system CIE-Lab where the L* value (brightness) ranges from black (0) to white (100), a* value ranges from green (-60) to red (+60) and the b* value ranges from blue (-60) to yellow (+60). The colorimeter was calibrated against a standard white reference tile. Samples were placed in a clear glass Petri dish (10 replicates), and colour measurements were done in triplicate. The chroma or saturation value (C*) and the hue angle (h°) were calculated by Eqs 1 and 2.


$$C^{*}=\sqrt{a^{{*}^{2}}+b^{{*}^{2}}}$$ (1)

$$\text{H}*={\text{tan}}^{-1}\left(\frac{a*}{b*}\right)$$ (2)

#### 2.3.2. Determination of major macronutrients and microntrients

Lyophilized *H*. *sabdariffa* aqueous extracts dissolved 10 mg/ml of in Nacl (0.1M) and ethanol (80%), respectively, were used for the evaluation of carbohydrate and protein contents. The total carbohydrate content was determined as described by Dubois et al., (1956) [11] using phenol (5%) with modifications. The absorbances were read at 630 nm with a spectrophotometer and the values obtained were directly extrapolated to a glucose standard curve (0–1 mg/ml; y = 0.0037x + 0.9904; R^2^ = 0.999). The protein content was determined by the Bradford method described by Kielkopf et al., (2020) [12] with some modifications. The absorbances obtained at 595 nm were directly extrapolated to a BSA standard curve (y = 1.3138x + 0.0119; R^2^ = 0.999). Total lipids were obtained using a Soxhlet extractor with hexane as solvent (NF V03-905). The potential energectic valuesof the different samples, was calculated using Atwater factors: = protein, carbohydrate and fat for 4, 4 and 9 kcal/100g, respectively. The mineral content (K, Ca, Mg, Na, Fe, Mn, Zn, Cu) of the different cakes was determined using a flame atomic absorption spectrophotometer. The content of these samples was determined using a calibration curve for each element measured.

### 2.4. Determination of bioactive nutrients content in the different extracts

#### 2.4.1. Vitamin C content

The method used for the quantification of ascorbic acid is the one described by Allan et al., (2017) [13] with minor modifications. This method is based on the decolourisation of 2,6-dichlorophenolindophenol (DCPIP) by ascorbic acid. To an aliquot of the extract (50 μL) was added 150 μL of DCPIP (0.2 mM). the absorbance is read with a spectrophotometer at 515 nm against a blank consisting of 150 μL DCPIP and 50 μL distilled water. A calibration curve is drawn with ascorbic acid in the concentration range 10 μg/mL to 100 μg/mL. Ascorbic acid contents are expressed in μg Ascorbic Acid Equivalent per 100 mg fresh leaves (μg EAA/100 mg dry matter).

#### 2.4.2. Phenolic contents

Total phenolic content of the extracts was determined by the method of Folin-Ciocalteau’s method [14] using gallic acid as standard. Total phenolic compounds were expressed as gallic acid equivalent per 100 g dry matter (gGAE/100 g, DM).

The total flavonoid content of the extracts was determined at 415 nm according to the method described by Meza et al., (2020) [15].

Quercetin was used for calibration curve and levels were expressed as mg quercetin equivalents (QE) per 100 g dry matter (gQE/100 g seeds dw).

#### 2.4.3. Hydrolysable tanins content

The determination of hydrolysable tannins was carried out according to the protocol of Mole and Waterman (1987) [16]. To an aliquot of the extract (5 mg/mL) was added3.5 mL of a solution prepared from 0.01 M FeCl_3_ in 0.001 MHCl). After 15 seconds, the absorbance of the mixture was read at 660 nm. The hydrolysable tannins were determined by the following formula 3:

$$\text{Proportion}\left(\%\right)=\left(\text{A}\;\text{x}\;\text{MW}\;\text{x}\;\text{V}\;\text{x}\;\text{DF}\right)/\varepsilon_{\text{mole}}\;\text{x}\;\text{W}$$ (3)

[17],

where A: absorbance; MW: molecular weight of gallic acid (170.12 g/mol); V: volume of extract used; DF: dilution factor; ε_mole_: 2169 mol/L (gallic acid constant); W: sample weight in g.

The powder of each extract 100 mg of the was vigorously shaken with 10 ml of 80% acetone for 1 min and centrifuged at 4500 rpm for 10 min. The absorbance of the supernatant was measured at 453, 505 and 663.

The results were expressed as mg ascorbic acid equivalent (AAE) per g dry extract (AAE /100 g).

### 2.5. Pigments content

#### 2.5.1. Determinationof β-Carotene and lycopene contents

β-Carotene, total chlorophyll and lycopene contents were determined according to the method described by Sombie et al. (2019) [17]. For this purpose, 100 mg of lyophilisates were dissolved in 5 ml of acetone/hexane (70/30) and β-carotene and lycopene contents were calculated according to the following Eqs 4 and 5:

$$\text{Lycopene}\left(\text{mg}/100\;\text{ml}\right)=-0.0458{\;\text{A}}_{663}+0.372{\;\text{A}}_{505}–0.0806{\;\text{A}}_{453}$$ (4)

$$\beta -\text{carotene}\left(\text{mg}/100\;\text{ml}\right)=0.216{\;\text{A}}_{663}–0.304{\;\text{A}}_{505}+0.452{\;\text{A}}_{453}$$ (5)

The powder (300 mg) of each extract was dissolved in 3 ml of 95% ethanol. The mixture was kept for 10 min in ice. After centrifugation for 1 min at 4500 rpm, the absorbance of the supernatant was measured at 665 nm and 649 nm for photosynthetic pigments using the formula 6:

$$\text{Total}\;\text{chlorophyll}\left(\mu \text{g}/\text{mL}\right)=6.1{\;\text{A}}_{665}+20.04{\;\text{A}}_{649}$$ (6)

where A_665_ = absorbance ate 665 nm andA_649_ = absorbance at 649 nm.

Results were expressed as μg/100 mg of fresh leaves extracts (g/100 g).

#### 2.5.2. Antocyanins contents

The total anthocyanin content of *H*. *sabdariffa* extract was quantified using the differential pH method [18].

Briefly, 1 mL of freeze-dried aqueous extract was mixed separately with 4 mL of each of the two buffers. The absorbance was measured at 510 and 700 nm with a after 15 min of incubation at room temperature. The results were expressed as follows: cyanidin-3-*o*-sambubioside (C3SE) equivalents per litre and the levels were obtained using the following Eq (7):

Totalanthocyanins=(AxMWxDFx1000)/ƐxL (7)

where

$$\text{A}:\text{Absorbance}=\left[\left({\text{A}}_{510}-{\text{A}}_{700}\right)\right]\text{pH}1.0-\left[\left({\text{A}}_{510}-{\text{A}}_{700}\right)\right]\text{pH}4.5;$$

MW: Molecular weight; DF: Dilution factor; Ɛ: Molar extinction coefficient of cyanidin-3-glucoside (26, 900 M^-1^ cm^-1^) and L = path-length (cm).

### 2.6. Determination of antioxidant activity

Three approaches were used to determine antioxidant activity: antiradical activity, lipid peroxidation inhibitory assay and assessment of superoxide dismutase activity.

#### 2.6.1. Antiradical activity

The first approach was to determine the percentage reduction indices of radical scavenging activity (RSA) and reactivity. The RSA (Eq 8) indicates the ability of the sample, at a given concentration, to reduce radicals and in many cases, increasing the concentration of the antioxidant leads to an increase in the relative indices. To eliminate the influence of concentration, the second approach is to estimate reactivity by determining the IC_50_ of each antioxidant. IC_50_ is the concentration (in mol/L) of DPPH corresponding to the change in optical density caused by a 50 ppm change in the antioxidant (Eq 9). The antioxidant capacity of compounds is higher, when IC50 is low. The IC50 is determined by the ratio of the absorbance of the reaction mixture containing the free radical and the antioxidant sample to the absorbance of the mixture without antioxidant (control solution) [19].

$$\%RSA=\frac{\left(Ac-Ae\right)*100}{Ac}$$ (8)

**A_C_**: absorbance of the control (DPPH)

**A_e_**: absorbance of the extract

IC50=(50PenteCDDPHCDO (9)

**C**_**DPPH**_**:** concentration of DPPH in mol/L.

**DO**_DPPH_: Absorbance of the control tube

#### 2.6.2. Lipid peroxidation inhibitory assay

The lipid peroxidation (LPO) inhibitory activity of the extracts was determined by the 2-thiobartbituric acid method [20]. FeSO_4_.7 H_2_O and H_2_O_2_ were used to induce peroxidation of egg lecithin. 0.2 mL of extract was mixed with 1 mL of egg lecithin (1%) in sodium phosphate buffer (50 mM, pH 7), then 50 μL of 0.5 mM FeSO_4_.7 H_2_O and 50 μL of 0.5 mM H_2_O_2_ were added. The resulting mixture is incubated at 37°C for 60 min. To 650 μL of the mixture is added 500 μL of 15% (w/v) trichloroacetic acid and 500 μL of 0.67% (w/v) 2-thiobarbituric acid and heated in boiling water for 15 min. The absorbances were read at 532 nm using a spectrophotometer. Quercetin is used as a positive control. The ability of the extracts to inhibit lipid peroxidation of lecithin is expressed as a percentage of inhibition according to the following formula 10:

$$\%of\;Inbition=\frac{\left(Ac-Ae\right)*100}{Ac}$$ (10)

**A**_**C**_**:** absorbance of the control (DPPH)

**Ae:** absorbance of the extract

#### 2.6.3. Assessment of superoxide dismutase activity

Each powder (500 mg) was dissolved in 5 ml of 50 mM sodium phosphate buffer pH 7.8. The supernatant was collected after centrifugation at 4 000 rpm for 10 minat 4°C to assess superoxide dismutase activity. The standard method described by Misra and Fridovich (1972) [21] was used for the determination of superoxide dismutase activity. This method is based on the inhibition of the epinephrine-adrenochrome transition by the enzyme. The mixture is obtained by adding 0.4 mL distilled water; 0.125 mL glacial ethanol; 0.075 mL chloroform; 0.1 mL 0.6 mM EDTA, 0.2 mL 0.25 M sodium carbonate and 0.1 mL 3 mM epinephrine to 0.25 mL extract. The mixture is shaken at 4°C for 5 min and then centrifuged at 4400 rpm for 10 min. The spectrophotometer reading was taken at 420 nm against a blank made under the same conditions with 0.25 mL of extraction buffer in place of the sample. The SOD activity was calculated according to Beer-Lambert law.

### 2.7. Statistical analysis

The Tukey’s test, the descriptive statistics and the creation of the various graphs were established using Excel; graphPad and XLSAT software version 2018. All measurement experiments were performed at least in triplicate.

## 3. Results and discussion

### 3.1. Physicochemical and biochemical characteristics

The physical and proximal composition, macronutrients and pigments content are presented in Table 1.

**Table 1:** Physics and proximate, major nutriments, pigments contents.

Parameters	Characteristics	CS	CA	GS	GA
Physics and proximate composition	pH	2.6 ± 0.3 a	2.36 ± 0.27a	5.87 ± 0,2 b	5.78 ± 0.3 b
	Humidity	6.68 ± 0.03 a	6.99 ± 0.02 a	7.07 ± 0,01 a	8.23 ± 0.04 a
	Total ash (g/100g)	7.72 ± 0.38 a	7.95 ± 1.17 a	6.05 ± 1.17 a	5.86 ± 2.44 a
	Titratable acid (mgEKOH/100g)	16.83 ± 5.39 a	42.5± 7.1 b	18.2 ± 1.74 b	22.16 ± 3.2 b
	L*	5.04 ±1.2 b	14.16±2.3 a	14.43± 1.5 a	11.627± 28 a
	a*	7.91± 1.2 a	3.46± 0.9 b	2.70± 1.0 b	2.06± 0.5b
	b*	2.40±0.6 c	9.61± 1.2 a	7.94± 0.2 b	7.44± 0.4 b
	c*	8.28± 1.4 a	10.22± 2.1 a	8.39± 1.7a	7.73± 1.2 a
	h*	17.56± 1.2 b	70.20± 0.8 a	71.18± 0.7 a	74.45± 0.8 a
Major nutriments	Total carbohydrates (g/100gMS)	7.51 ± 1.18 b	4.60 ± 0.87 c	12.10 ± 0,5 a	12.44 ± 1.11 a
	Total protein (g/100gMS)	11.96 ± 0,2 a	11.26 ± 0.3 a	12.32± 1,8 a	10.64± 1.68 a
	Total lipids (g/100gMS)	3.29 ± 0,58 b	3.24 ± 0.59 b	20.69 ± 1,8 a	18.80 ± 3.2 a
	Energy balance (KJ)	107.49 ± 5.94 a	92.6 ± 5.74 a	283.99 ± 23,72 b	261.8 ± 35.64 b
Pigments	B-carotène (g/100gMS)	0.53 ± 0.27 a	0.32 ± 0.17 a	0.01 ± 0.01 b	0.01 ± 0.00 b
	Lycopène (g/100gMS)	0.85 ± 0.34 a	0.19 ± 0.1 b	0.19 ± 0.65 b	0.03 ± 0.01c
	Chlorophyll (g/100gMS)	4.12 ± 1.04 a	3.22 ± 0.95 a	2.92 ± 0.8 ab	1.19 ± 0.95 b
	Anthocyans (g/100g)	1.73 ± 0.09a	0.19 ± 0.02 b	0.04 ± 0,04 b	0.02 ± 0.19 b

Values with similar letters within row are not significantly different at p ≤ 0.05.

The physico-chemical parameters were influenced by the variety and the nature of the sample. Indeed, the calyxes of the two varieties (*sabdariffa and altrissima*) showed respectively an acidic pH of 2.6 ± 0.3 and 2.36 ± 0.27 compared to the seeds. The *altrisssima* variety also showed the highest titratable acidity in calyxes (42 ± 7.1 mgKOH.100g^-1^MS) and seeds (22.16 ± 3.2 mgKOH.100g^-1^MS) respectively compared to the *sabdariffa* variety. These results corroborate those obtained by Bothon et al., (2020) [22] in *Hibiscus sabdariffa* seeds of 23.10 ± 0.22 mgKOH.100g^-1^MS and 18.20 ± 0.40 mgKOH.100g^-1^MS respectively. This high titratable activity of the *altrissima* phenotype supports its traditional use as a potash in tea preparation.

This high titratable activity of the *altrissima* phenotype supports its traditional use as potash in tea preparation. No significant differences were observed for ash content and moisture. The total mineral content was similar to that obtained in calyxes which were 9.04 ± 0.4 g 100 g MS^-1^ and 10.10 ± 0.38 g. = 100 g^-1^ MS for red *(sabdariffa*) and white (*altrissima*) phenotypes respectively. These results are similar to those obtained by Ahmed et al, (2019) [23]. In addition, the highest L* light intensity was recorded in the calyxes (14.43± 1.5) of the *altrissima* variety and the lowest in the calyxes of the *sabdariffa* variety. The highest intensity of red colouring, assessed by the a* value, was observed in red calyxes. It was significantly different from the other samples. The chromaticity index C*, which measures colour saturation was similar in the different samples (CA, GA and GA), except for the calyxes of the *sabdariffa* variety. The red coloration of the calyxes of the *sabdariffa* phenotype shows the presence of bioactive compounds including the flavyliun cation which turns red in acidic medium and the saturation may be related to the anthocyanin concentration [24].

The biochemical characterisation in macronutrients was influenced by the nature of the sample. Indeed, seeds from both phenotypes showed the best carbohydrate and lipid contents while no significant difference was observed for protein composition. Similar proportions were obtained for carbohydrate content (101.1 ± 11.3 g.kg^-1^) and protein content (92.5 ± 4.3 g.kg-1) in red calyxes respectively [24]. lipids content of the samples was higher than those obtained elesewhere [24]. This difference could be explained by the climatic parameters including solar temperature. The biochemical characterisation of the macromolecules shows the nutritional and energetic quality of the extracts, adapted to the satisfaction of human needs of human needs. Moreover, the amino and fatty acids resulting from the degradation of proteins and fats are vectors of antihypertensive activities [25].

Phenotype and geographical sample origin of the sample impacted pigment contents. Calyxes of the red phenotype showed the highest content of anthocyanins (1.73 ± 0.09 mg.100g^-1^), β-carotene (0.53 ± 0.27 mg.100g^-1^), lycopene (0.85 ± 0.34 mg.100g^-1^) and chlorophyll (4.12 ± 1.04 mg 100 g^-1^). The results corroborate those of Wong et al., (2002) [26] on the presence of carotenoids (lycopene and betacarotene) and flavonoids (anthocyanins) in our extracts and these constituents influence the red coloration of the extracts. According to Wong et al., 2002, the red colouring of the calyxes of the *sabdariffa* phenotype is related to their high concentration of these pigments. Moreover, these pigments play an important role in the control of oxidative stress.

**Fig 2:**
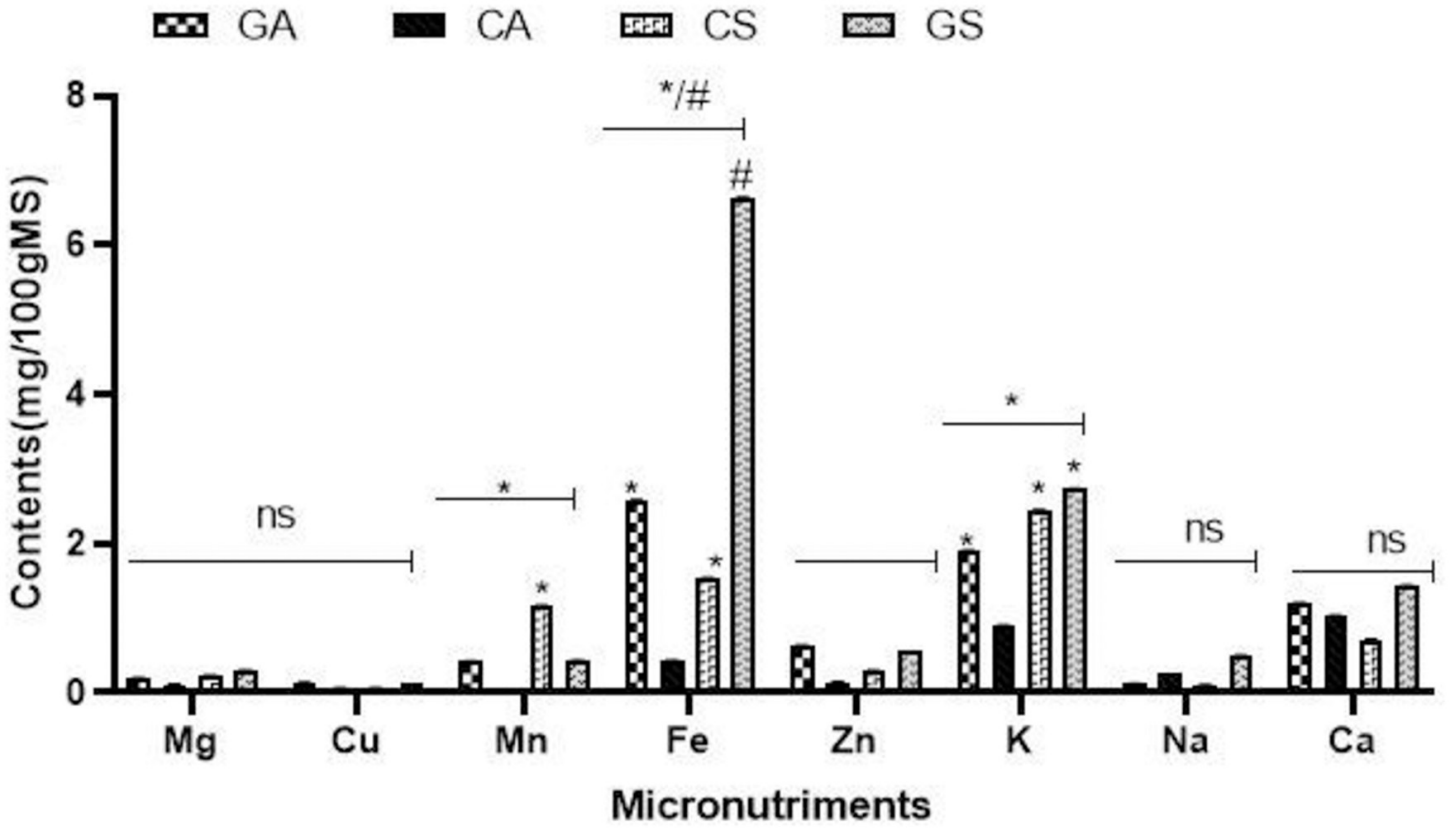
Micronutriments content. Histograms with the same symbols are not significantly different p<0.05(*); p<0.01 (^#^).

The samples showed interesting mineral contents that varied significantly according to the variety (Fig 2). Indeed, the seeds of the *sabdariffa* variety showed the best contents of Ca, Na, K, Fe, Cu, while the calyx of this same variety showed the highest content of Mn. The analysis shows a high iron content (6.64 mg 100g^-1^) and a low copper content (0.13 mg 100g^-1^). The results are higher than those obtained by Parkouda et al., [1] and lower than those obtained by salami et al. [27] the other varieties. The order of mineral content in the seed is as follows:: Fe >K >Ca > Zn > Na> Mn > Mg >Cu, which differs from data obtained by Parkouda (Fe > Zn > Na >Ca >P >K) [1]. This difference could be explained by the nature of the cultivation soils and the mechanism of mineral absorption by the plants. Natural variation in primary metabolites tends to be controlled by a large number of loci that influence phenotypic and nutritional traits [28]. In addition to the nutritional contribution of these micronutrients: Fe, Na and K; they are also involved in several biological processes (antiradical activity, antihypertensive and diuretic activity) [25].

### 3.2. Bioactive nutrients content

**Fig 3:**
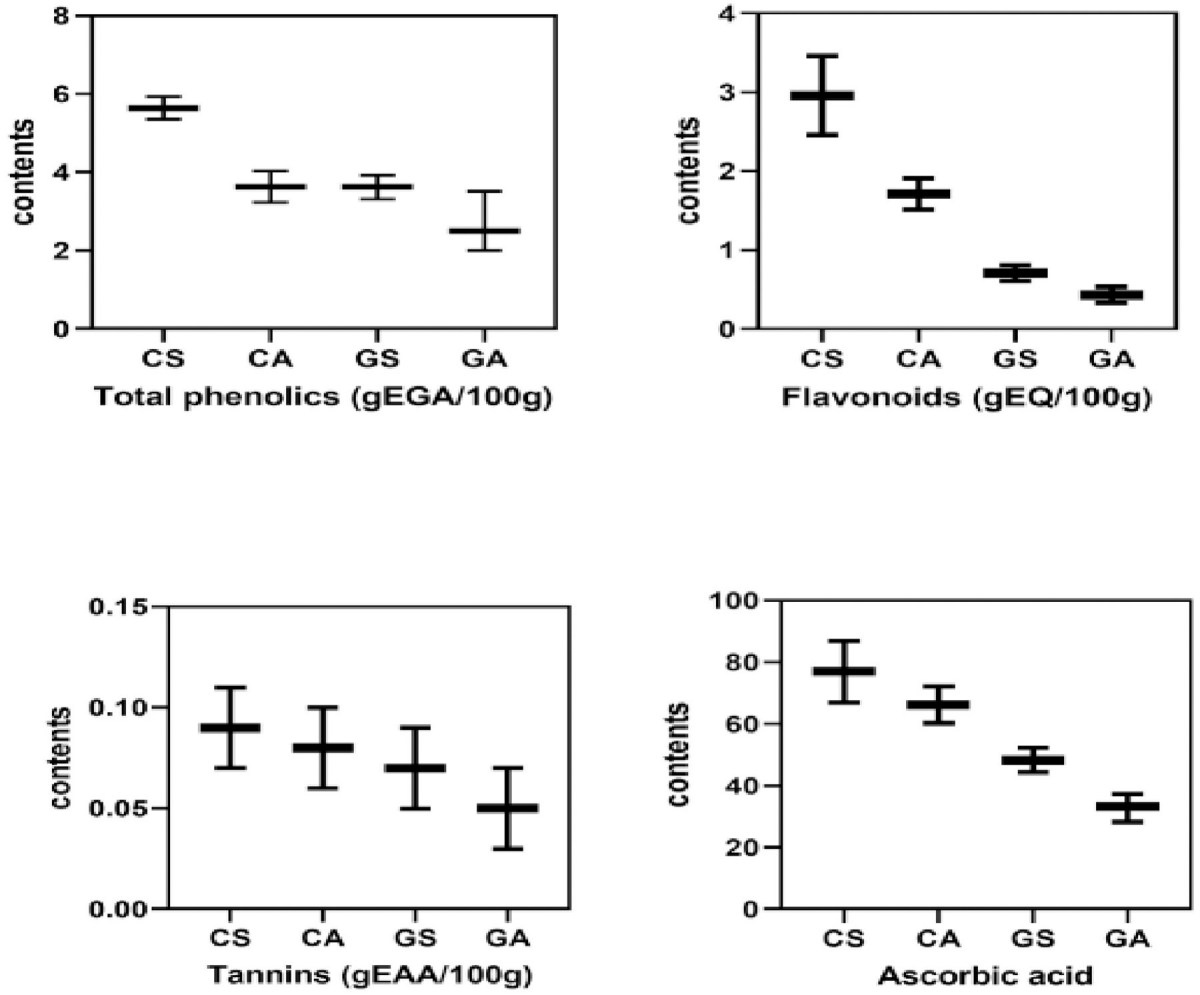
Comparative study of bioactive coumpounds contents.

**Fig 4:**
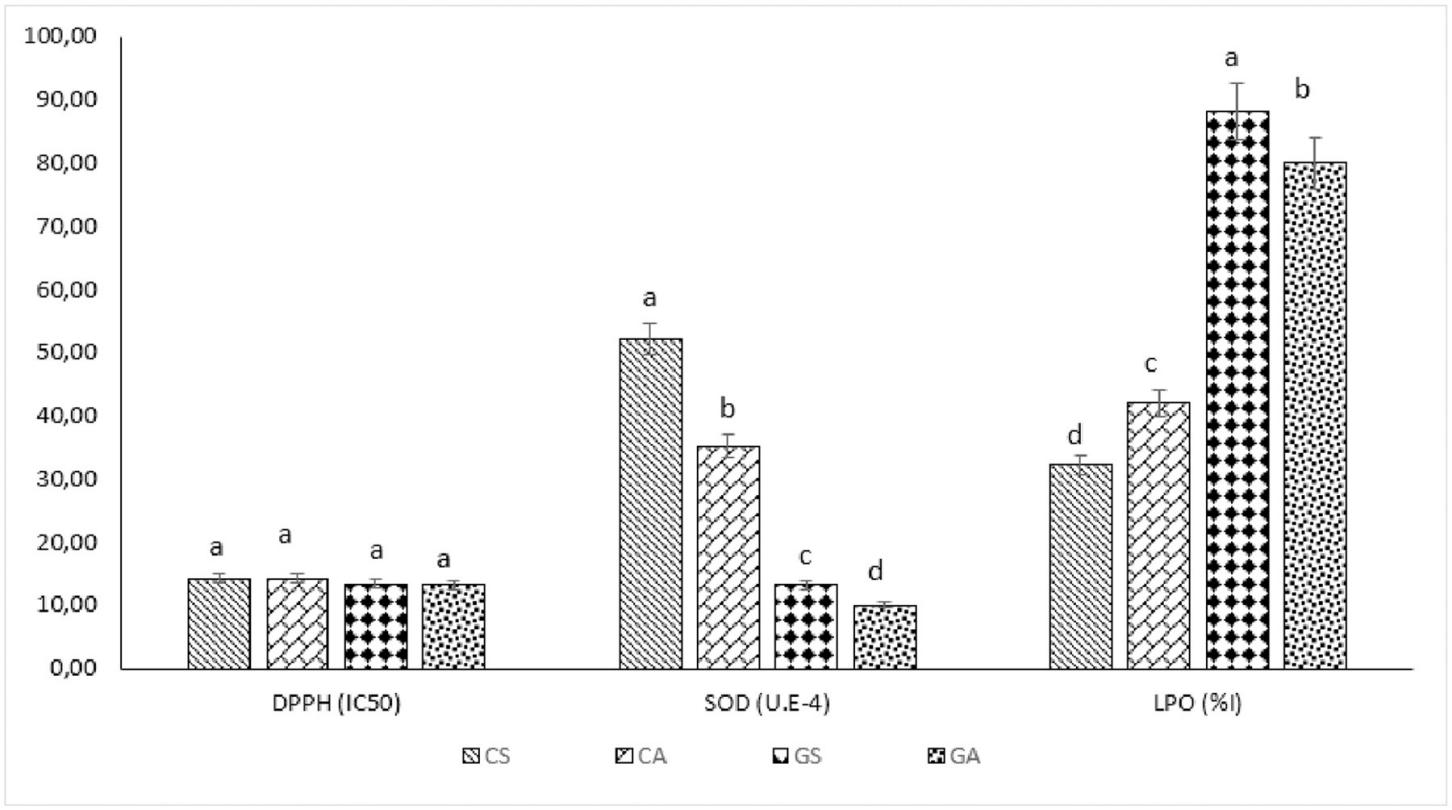
Antioxidents activities of extract *Hibiscus sabdariffa*. Values with similar letters within row are not significantly different at p ≤ 0.05.

Analysis (Fig 3) showed that the calyxes of the *sabdariffa* phenotype had the highest proportions of total phenolics (5.64 ± 10.5 gEGA.100g^-1^), flavonoids (2.011 ± 0.95 gEQ.100g^-1^), tannins (0.091 ± 0,06 gEAA.100g^-1^) and ascorbic acid (76.92 ± 10.75 mgEAA.100g^-1^). The results are similar to those of Deli et al., [29] which obtained different levels of total phenolics (4.22 ± 0.2 mgEGA.100g^-1^) and flavonoids (2.16 ± 0.2 mgER.100g^-^) and condensed tannins (20.8 ±0.2EC). The levels of ascorbic acid were higher than those found by Salami and Afolayan [27]. The same holds true for phenolic contents (Fig 4). This difference could be explained by the effect of water and salt stress to which our plants were exposed. In addition, calyxes have more bioactive compounds than seeds. This difference could be justified by the mechanism of synthesis of phenolic compounds. Indeed, when subjected to biotic stress, plants produce secondary metabolites in order to protect themselves. Thus, the seed is less exposed to aggressors compared to the calyxes which are the protective organs of the plant [30].

Several methods are used to evaluate potential antioxidant activities. Indeed, the Zn-Cu-SOD complex is the major antioxidant enzyme present in intracellular fluids and is usually expressed in blood vessels, while an accumulation of LPO products in human tissues is a major cause of tissue and cell dysfunction. It plays an important role in oxidative stress-related diseases. Determination of SOD activity and LPO inhibition is part of the indirect determination of ROS activity and chronic diseases including hypertension. The red phenotype has the best superoxide dismustase activity for calyxes and the best lipid peroxidation inhibitory activity for seeds, respectively. These results are similar to those of Usoh et al, (2005) [31]. Indeed, these antioxidant systems show that screened extracts could play a fundamental role in cellular defense against free radicals [8]. The different extracts exhibited similar antiradical powers. The free radical scavenging capacity of the extracts were better than those obtained by Deli et al., (2020) [32] on *H*. *sabdariffa* fractions according to size (46.00 μg ml^-1^ ± 3.46 μg ml^-1)^ for a particle size of 0–180 um. This difference could be explained by the pretreatment and used extraction methods.

### 3.3. Effect of season and production area on bioactive compounds

Season and area of production environmental factors can influence the phytochemical and nutritional profile of plants [33]. There is noticeable effect of season on the protein, carbohydrate, total phenolic, flavonoid and IC50 (DPPH) content of extracts (Table 2). From the analysis, it can be seen that season 2 (December 2020) had significantly different contents of carbohydrates, total phenolics, flavonoids and free radical scavenging activity compared to 2019. Water stress induces a strong synthesis of biomolecules. Indeed, Bobo-Dioulasso area had an average rainfall of 256.8 mm and 234.0 mm in 2019 and 2020, respectively. This could in part justify the difference in biochemical composition between years 2020 compared to 2019. There is also the influence of the production area on the synthesis of bioactive molecules (Table 3). However, there is no significant difference in the content of protein, carbohydrates, total phenolics, flavonoids and IC50 (DPPH) in our extracts. The climatic similarity of its production areas would limit the influence of water stress on the synthesis of biomolecules. Environmental conditions such as temperature, precipitation, photoperiodicity, relative humidity govern the metabolite composition as well as the genetic characteristics of the plant [34].

**Table 2:** Effect of season on bioactive compounds.

Parameters		CS	CA	GS	GA
Proteins (g/100gMS)	Saison 1	11.96 ± 0.2 a	11.26 ± 0.3 a	12.32± 1.8 a	10.64± 1.68 a
	Saison 2	11.54 ± 2.07a	11.42 ± 0.7a	12.86 ± 0.92a	9.61 ± 0.55a
Carbohydrats (g/100gMS)	Saison 1	1.26 ± 0.23 b	1.22 ± 0.17b	0.81 ± 0.07 b	0.75 ± 0.03 b
	Saison 2	6.48 ± 1.06 a	7.42 ± 0.17 a	9.33 ± 0.35 a	9.29 ± 0.58 a
Total phenolics (gEAA/100gMS)	Saison 1	5.64 ± 10.5 b	3.93 ± 0,4 b	3.62 ± 1.0b	2.50 ± 0.5 b
	Saison 2	14.61 ± 2.12 a	12.56 ± 2.4 a	11.95 ± 1.7	5.2 ± 1.2 a
Flavonoids (gEQ/100gMS)	Saison 1	2.01 ± 0.95 b	1.26 ± 0.14 a	0.77 ± 0.65 a	0,33 ± 0.20 a
	Saison 2	4.4 ± 0.2 a	1.9 ± 0.2 a	0.92 ± 0.43a	0.21± 0.1 a
IC_50_ (DPPH) μg.ml^-1^	Saison 1	1.43± 0.1 b	1.42 ± 0.23 b	1.33 ± 0,31 b	1.3 ± 0.2 b
	Saison 2	1.85 ± 0.2 a	2.3 ± 0.25 a	2.5 ± 0,2 a	2.4 ± 0.2 a

Values with similar letters within row are not significantly different at p ≤ 0.05.

**Table 3:** Effect of production areas on bioactive compounds.

		Bobo Dioulasso	Dano	Nouna
Total	CS	6.55 ± 1.2^a^	6.52 ±0.9 ^a^	5.36 ± 1.9 ^a^
	CA	3.50 ± 0.5 ^ab^	4.00 ± 0.8 ^a^	3.28 ±1.2 ^a^
Phenolics	GS	3.31 ±1.7^ab^	4.13 ± 0.3 ^a^	2.83 ±0.8 ^a^
	GA	2.46 ± 0.3 ^ab^	2.45 ± 0.2 ^a^	2.55 ± 0.4 ^a^
Flavonoïds	CS	0.84 ±0.2 ^a^	0.82 ± 0.5 ^a^	0.66 ± 0.4 ^a^
	CA	0.33 ± 0.1 ^a^	0.37 ± 0.04 ^a^	0.29 ± 0.02 ^a^
	GS	2.23 ± 0.2 ^a^	2.22 ± 0.1 ^a^	1.86±0.2 ^a^
	GA	1.27±0.3 ^a^	1.31 ±0.3 ^a^	1.21 ± 0.2 ^a^
Tanins	CS	0.12 ±0.3 ^a^	0.09 ±0.2 ^a^	0.05 ±0.3 ^a^
	CA	0.07 ± 0.2 ^a^	0.06 ±0.02 ^a^	0.03±0.2^a^
	GS	0.06±0.1 ^b^	0.08±0.2 ^ab^	0.09 ±0.1 ^a^
	GA	0.13±0.5 ^a^	0.02 ± 0.2^b^	0.05±0.4 ^ab^
Anthocyanins	CS	1.89 ±0.2 ^a^	1.80 ±0.3 ^a^	1.52 ±0.5 ^a^
	CA	0.04 ±0.3 ^a^	0.02 ±0.1 ^a^	0.01±0.05^a^
	GS	0.03 ± 0.15 ^a^	0.05 ± 0.3 ^a^	0.02 ± 0.1 ^a^
	GA	0.05 ± 0.2 ^a^	0.04 ± 0.2^a^	0.05±0.1 ^a^

Values with similar letters within row are not significantly different at p ≤ 0.05.

### 3.4. Principal Component Analysis (PCA)

**Fig 5:**
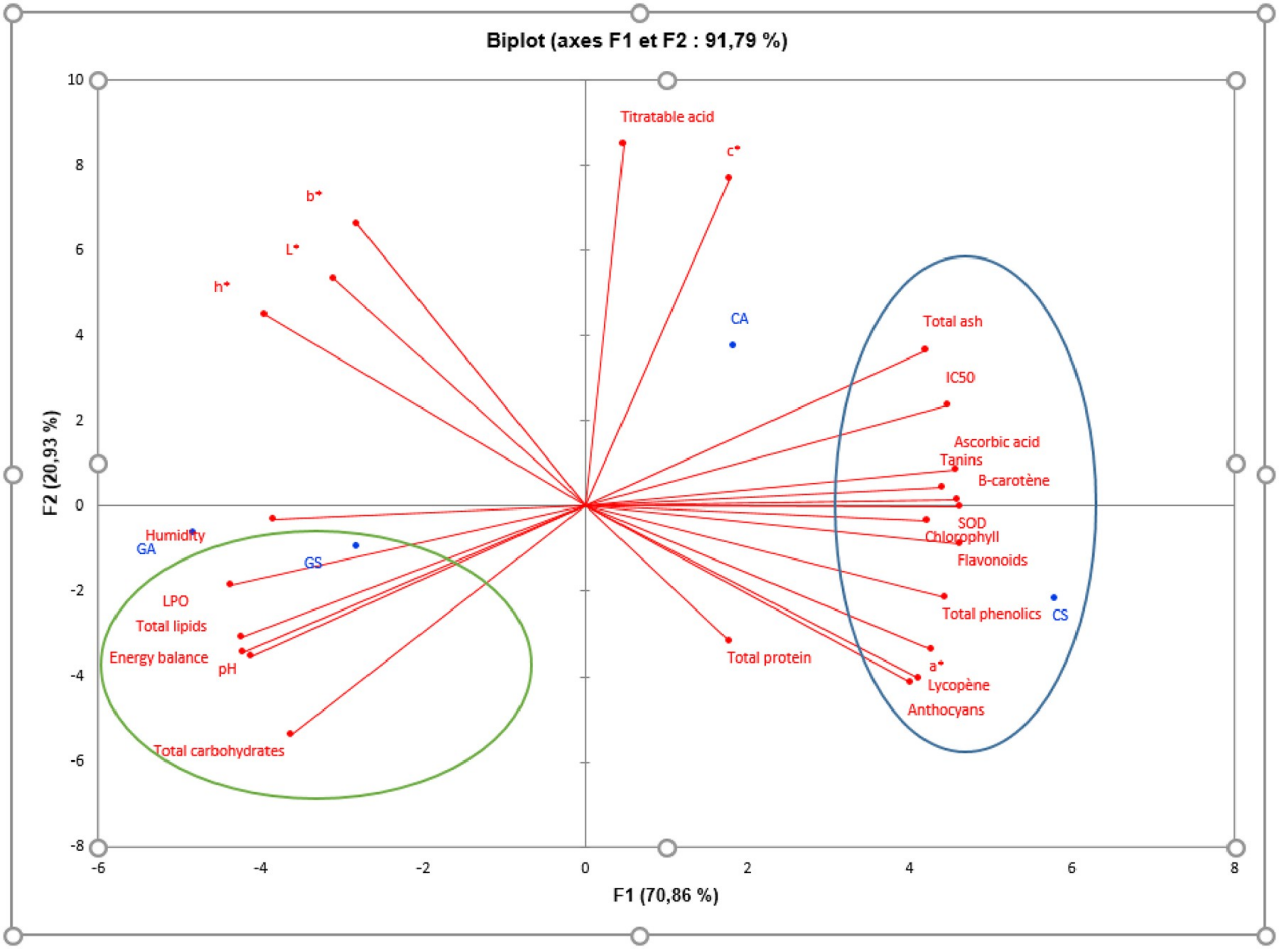
Principal Component Analysis (PCA).

PCA is a statistical analytical tool that explains the variance of large intercorrelated variables by transforming them into a smaller set of independent and uncorrelated principal components. In our study, PCA presents 91.79% of the variance of biochemical attributes (macromolecules, phytochemical profile and antioxidant activity) in samples of *H*. *sabdariffa* around two axes (Fig 5). The highest component loadings were observed for total phenolics, flavonoids, anthocyanins, chlorophyll, reddish colour, free radical scavenging activity and SOD in calyxes of the red phenotype (CS), while its seeds (GS) were recorded as maximum loadings, carbohydrate content, lipid content, LPO inhibitory activity and pH. The calyxes of the white phenotype (CA) showed the presence of a maximum concentration of tritratable acidity. These results support the existing positive correlation between colour, phenolic composition, antioxidant activity and medicinal properties [35]. The redness of the calyxes (CS) can be considered as a visual indicator in content in phenolic compounds and potential bioactivity.

## 4. Conclusion

Varietal differences and seasonal variation in the nutritional and antioxidant composition of two *H*. *sabdariffa* accessions were assessed. The influence of genetic variability and season on nutritional and phenolic traits was demonstrated. Positive correlations between nutritional traits, phenolic traits, antioxidant activity and colour were observed. From the principal component analysis, it is clear that the red phenotype calyxes have both nutritional and medicinal properties.

## Supporting information

S1 Data (XLSX)
