# Correction: *Hibiscus sabdariffa*: Genetic variability, seasonality and their impact on nutritional and antioxidant properties

**DOI:** 10.1371/journal.pone.0295299

**Published:** 2023-11-30

**Authors:** Abdoudramane Sanou, Kiessoun Konate, Roger Dakuyo, Kaboré Kabakdé, Hemayoro Sama, Mamoudou Hama Dicko

The fourth author’s name is spelled incorrectly. The correct name is: Kaboré Kabakdé. The correct citation is: Sanou A, Konate K, Dakuyo R, Kabakdé K, Sama H, Dicko MH. (2022) *Hibiscus sabdariffa*: Genetic variability, seasonality and their impact on nutritional and antioxidant properties. PloS ONE 17(3): e0261924. https://doi.org/10.1371/journal.pone.0261924

There is an error in affiliation 1 for authors Abdoudramane Sanou, Kiessoun Konate, Roger Dakuyo, Kaboré Kabakdé, and Mamoudou Hama Dicko. The correct affiliation is: Laboratory Biochemistry, Biotechnology, Food Technology and Nutrition (LABIOTAN), Department of Biochemistry and Microbiology, University Joseph KI-ZERBO, 03 B. P. 7021, Ouagadougou, Burkina Faso.
